# Gypenosides Prevent H_2_O_2_-Induced Retinal Ganglion Cell Apoptosis by Concurrently Suppressing the Neuronal Oxidative Stress and Inflammatory Response

**DOI:** 10.1007/s12031-019-01468-9

**Published:** 2020-01-02

**Authors:** Hong-Kan Zhang, Yuan Ye, Kai-Jun Li, Zhen-ni Zhao, Jian-Feng He

**Affiliations:** Department of Ophthalmology, the First Affiliated Hospital of Guangxi Medical University, Nanning, 530021 Guangxi Zhuang Autonomous Region China

**Keywords:** Gypenosides, Retinal ganglion ceils, Oxidative damage, Neuroprotection

## Abstract

Our previous study demonstrated that gypenosides (Gp) exert protective effects on retinal nerve fibers and axons in a mouse model of experimental autoimmune optic neuritis. However, the therapeutic mechanisms remain unclear. Thus, in this study, a model of oxidative damage in retinal ganglion cells (RGCs) was established to investigate the protective effect of Gp, and its possible influence on oxidative stress in RGCs. Treatment of cells with H_2_O_2_ induced RGC injury owing to the generation of intracellular reactive oxygen species (ROS). In addition, the activities of antioxidative enzymes decreased and the expression of inflammatory factors increased, resulting in an increase in cellular apoptosis. Gp helped RGCs to become resistant to oxidation damage by directly reducing the amount of ROS in cells and exerting protective effects against H_2_O_2_-induced apoptosis. Treatment with Gp also reduced the generation of inducible nitric oxide synthase (iNOS) and cyclooxygenase-2 (COX-2), and increased nuclear respiratory factor 2 (Nrf-2) levels so as to increase the levels of heme oxygenase-1 (HO-1) and glutathione peroxidase 1/2 (Gpx1/2), which can enhance antioxidation in RGCs. In conclusion, our data indicate that neuroprotection by Gp involves its antioxidation and anti-inflammation effects. Gp prevents apoptosis through a mitochondrial apoptotic pathway. This finding might provide novel insights into understanding the mechanism of the neuroprotective effects of gypenosides in the treatment of optic neuritis.

## Introduction

Optic neuritis (ON) is a common neuro-ophthalmologic inflammatory disease that results in persistent vision impairment in young adults. Idiopathic demyelinating optic neuritis (IDON), the most common clinical type of ON, is strongly associated with multiple sclerosis (MS). However, the pathogenesis of MS or IDON remains largely obscure. MS is thought to be an autoimmune disease characterized by the CD4^+^ T cell-mediated demyelination in the central nervous system. CD4^+^ T cell activation causes an inflammatory cascade reaction and oxidative stress, which produces a large number of reactive oxygen species that utilizes endogenous antioxidant enzymes. Thus, in acute or chronic MS and ON, a wide range of demyelinating lesions occur, along with axonal loss and neuronal apoptosis leading to irreversible loss of vision. Corticosteroids are commonly used in the treatment of ON, the pharmacological effects of which are based on reducing inflammation, not neuroprotection. Therefore, they have limited effects on improving the prognosis of visual acuity. One study even indicated that methylprednisolone accelerated neuronal apoptosis in the central nervous system. Patients with ON urgently need a novel medication with anti-inflammatory properties as well as neuroprotective effects in order to improve their visual prognosis. Gypenosides, the saponin extracts from the plant *Gynostemma pentaphyllum*, have been shown to provide various bioactivities such as antioxidation, hepatoprotection, antilipidemia, and inflammation reduction. Importantly, neuroprotective effect of Gp has been proven, and current research is mainly focused on rescue of ischemia-reperfusion injury, Alzheimer’s disease, and Parkinson’s disease. However, it needs to be established whether Gp has a protective effect in ON. Our previous studies have demonstrated neuroprotective effects of Gp in experimental autoimmune optic neuritis (EAON). We generated the hypotheses that Gp may have therapeutic activity in ON, a neurodegenerative disease caused by autoimmune demyelination. In the present study, a retinal ganglion cell (RGC)-based oxidative damage model was employed to evaluate the potential therapeutic antioxidant, anti-apoptosis, and neuroprotection effects of Gp on RGCs.

## Materials and Methods

### Animals

Sprague Dawley rats (5–6 days old) were purchased from the Guangxi Medical University Laboratory animal center (Nanning, China). All animal procedures were in strict accordance with the association for research in vision and ophthalmology (ARVO) statement.

### High-Performance Liquid Chromatography Analysis

High-performance liquid chromatography (HPLC) was used to characterize Gp on an InertSustain C18 LC Column (2 μm, 3.0 × 150 mm; GL Sciences Inc.) with the Agilent 1290 Infinity II. The mobile phase consisted of acetonitrile and phosphorous acid solution (0.1% in water), with a gradient elution of 10–95% acetonitrile between 0 and 42 min. The column temperature was maintained at 35 °C. The flow rate was 0.35 mL/min, the detection wavelength was 203 nm, and 20 μL of a methanol solution of Gp was used for analysis. Two typical standard substances, Gp-17 and Ginsenoside Rb1 (Chengdu Mansite Biotechnology Co., LTD., Chengdu, Sichuan province, China), were also analyzed at the same condition to aid in the characterization of Gps.

### Culture of Purified RGCs

To isolate RGCs, rats were anesthetized with an overdose of pentobarbiturate, and their retinae were dissected. The tissues were incubated in Dulbecco’s phosphate buffered saline (DPBS) containing 15 units/ml papain, 70 U/ml collagenase, and 0.04% DNase for 30 min at 37 °C. The tissues were then sequentially triturated in D-PBS containing 0.15% bovine serum albumin (BSA), 0.04% DNAse. Cells were resuspended in D-PBS and the upper layer of cell suspension collected after allowing the suspension to stand for 2 min. The retinal cell suspension was centrifuged at 120*g* for 5 min, and the pellet was resuspended in DMEM/F12 panning buffer (DPBS, 0.02% BSA, 5 μg/ml insulin) and plated in 6-well plates coated with anti-macrophage antibody (1:100) to remove microglial cells for 30 min. The plates were shaken lightly every 10 min. The non-adherent cells were harvested and incubated for 1 h at room temperature on 6-well plates coated with rat anti-mouse Thy 1.1 antibody (1:100) and shaken lightly every 30 min. Non-adherent cells were washed away and the bound cells were resuspended in serum-free neurobasal medium containing 2% B27 supplement, BDNF (40 ng/ml), and CNTF (40 ng/ml), and seeded at a density of 1 × 10^5^ cells/well onto poly D-lysine and laminin-coated coverslips placed in 6-well plates. Half of the media was changed every third day. RGCs were cultured for 5 days at 37 °C, in 5% CO2 with 95% humidity.

### Toxic Insults and Drug Treatment

To induce oxidative stress, RGCs were exposed to 100 μM H_2_O_2_ for 4 h. The cells in the Gp treatment group were treated with different concentrations of Gp (50, 100, and 200 μg/ml) for 4 h followed by treatment with H_2_O_2_. Gp (Chengdu Mansite Biotechnology Co., LTD., Chengdu, Sichuan province, China) was prepared in DMSO and diluted with fresh complete medium immediately before use such that final concentration of DMSO in serum-free neurobasal medium was 0.1%. The control cells were also treated with DMSO at the 0.1% final concentration in serum-free neurobasal medium. For the detection of PI3K/Akt signaling pathway, we set up a blank control group, oxidative damage group (with or without PI3K/AKT inhibitor LY294002), and the 100 μg/ml Gp group (with or without PI3K/AKT inhibitor LY294002). For the suppression of the PI3K pathway, RGCs were pretreated with LY294002 (10 uM) for 30 min. After this, they were incubated with Gp or H_2_O_2_.

### Assessment of Cell Viability

Cell survival was estimated by the MTT assay. RGCs were cultured in 96-well plates. Briefly, after treatment for 4 h, the culture medium was removed and replaced the fresh medium containing 5 mg/mL MTT and incubated for another 4 h at the same condition. Then, the medium was discarded, and 150 μl DMSO was added to each well with shaking for 10 min. The optical density (OD) was detected at 490 nm using a microplate reader.

### ROS Concentration Assay

Intracellular ROS levels were assessed using DCFH-DA probe (Sigma, USA). RGC cultures were digested using a trypsin containing cocktail of protease inhibitors (Sigma, USA). Then, the cell suspension was centrifuged within D-Hanks for 5 min at 1500 rpm. After centrifugation, the supernatant was discarded. The cell suspension was treated with DCFH-DA at a final concentration of 10 μM for 30 min at 37 °C in the dark. After incubation, the cells were washed and analyzed using flow cytometry (BD Biosciences) and compared with a sham group. Data were analyzed using the FCSExpress V3 program (DeNovo Software, Los Angeles, CA).

### Apoptosis Detection Using Hoechst 33342 Staining

After treating with H_2_O_2_, the cells were washed with PBS and stained with Hoechst 33342 (5 μg/mL) for 20 min at 37 °C in the dark. Images were obtained using a fluorescence microscope (Nikon Instruments Inc., Melville, NY, USA) at × 100 magnification. Ultraviolet excitation wavelengths at 346 nm were used to obtain images of nuclei labeled with Hoechst-33342. The cell with pyknotic nuclei was identified as apoptotic cells. Images from five randomly selected fields were taken from each well, and the number of apoptotic or total cells were counted. The percentage of pyknotic nuclei in total was calculated, and then averaged in each well in order to determine the proportion of apoptosis in each group.

### Apoptosis Detection Using TUNEL Staining

To confirm the results of the Hoechst 33342 staining, apoptosis was assayed using a TUNEL stain.

For the assessment of apoptosis, primary cultured RGCs grown on slides were rinsed once with PBS and fixed in freshly prepared 4% paraformaldehyde in PBS for 60 min. The slides were rinsed twice with PBS. Cells were permeabilized by incubating in a permeabilization solution containing 0.1% Triton X-100 and 0.1% sodium citrate for 2 min on ice. Cells were incubated with 50 μl of TUNEL reaction mixture for 30 min at 37 °C in a dark and humidified atmosphere. Slides were washed three times with PBS. TUNEL staining was observed under a fluorescence microscope, used an excitation wavelength in the range of 450–500 nm and detection in the range of 515–565 nm (TE2000U, Nikon, Tokyo, Japan), and photographed using the Image-Pro Plus 6.0 imaging system. Before taking photomicrographs, Hoechst 33342 was added to count the total cell number. Apoptotic cells showed green fluorescence. Five random fields were counted in each well, and three wells were selected from every treatment groups. The percentage of cell death was determined as the ratio of the number of TUNEL positive cells to the total number of Hoechst 33342 stained cells.

### Quantitative RT-PCR

Total RNA was extracted from RGCs from each treatment group using Trizol (Invitrogen) and chloroform, and was purified and quantified for reverse transcriptions. Quantitative real-time PCR was performed using the Bio-Rad iQ5 system using Bio-Rad proprietary iQ5 software. The results were calculated with the ∆Ct method. The relative gene expression was normalized to an internal control of actin. Predesigned primers and probes were purchased from LifeTec. Primer sequences of target genes are shown in Table 1.

**Table 1:** Primer sequences of target genes

Genes of interest	Primer sequences
Sagene-actin-F	CCCATCTATGAGGGTTACGC
Sagene-actin-R	TTTAATGTCACGCACGATTTC
QPCR-heme oxygenase 1-F	AGGGAAGGCTTTAAGCTGGTG
QPCR-heme oxygenase 1-R	GTGGGGCATAGACTGGGTTC
QPCR-COX2-F	GCTCAGCCATGCAGCAAATC
QPCR-COX2-R	GGGTGGGCTTCAGCAGTAAT
QPCR-caspase 3-F	GGAGCTTGGAACGCGAAGA
QPCR-caspase 3-R	ATCGGTACCATTGCGAGCTG
QPCR-Gpx1-F	TTCGGACATCAGGAGAATGGC
QPCR-Gpx1-R	GGAATGCCTTAGGGGTTGCT
QPCR-iNOS-F	TGGTGAGGGGACTGGACTTT
QPCR-iNOS-R	TGTTGGGCTGGGAATAGCAC
QPCR-Nrf2-F	GCCCTCAGCATGATGGACTT
QPCR-Nrf2-R	ATGTGGGCAACCTGGGAGTA
QPCR-BAX-F	TTGCTACAGGGTTTCATCCAGG
QPCR-BAX-R	CACTCGCTCAGCTTCTTGGT
QPCR-BCL-2-F	CACCCCTGGTGGACAACATC
QPCR-BCL-2-R	TAGTTCCACAAAGGCATCCCAG

Data were analyzed using the SDS 1.9.1 software (Applied Biosystems). All experiments were performed in triplicates and were averaged to obtain the data point for each specimen.

### Immunoblot Analysis

An automated protein immunoblot was performed using Simple Wes Wb (ProteinSimple, San Jose, CA) for protein detection. After treatment, RGCs were washed with ice-cold PBS, scraped, and then incubated in lysis buffer containing RIPA (Beyotime Institute of Biotechnology, Shanghai, China), supplemented with protease phosphatase inhibitors (Roche Pharmaceutical Ltd., Basel, Switzerland) on ice for 10 min. The lysate was centrifuged at 6000 rpm for 5 min at 4 °C and the supernatant was collected. Total protein concentration of the supernatant was determined by BCA microplate assay (Pierce BCA Protein Assay Kit) according to manufacturer’s instructions. The protein concentration of each sample was adjusted to 0.5 μg/μl and the sample loaded according to manufacturer’s instructions. Primary antibodies are as follows: iNOS (Genetex, USA), COX-2 (abcam, UK), Nrf-2 (abcam, UK), heme oxygenase-1 (abcam, UK), GPx-1/2 (Santa, USA), Akt (pS472/pS473) (BD PMG, USA), Bcl-2 (BD PMG, USA), Bax (BD PMG, USA), caspase-3 (CST, USA), GAPDH (abcam, UK)*, anti-rabbit secondary antibody (Proteintech, PRC). All the primary antibodies were diluted 1:50 and secondary antibody diluted 1:100. The proteins were quantified and analyzed with Compass software (ProteinSimple, San Jose, California, USA). Values of each index were normalized to GAPDH to calculate relative protein expression levels.

### Statistical Analyses

Data are presented as mean ± standard error (SE). Three biological replicates were carried out for such of the experiments. Statistical analysis of results was performed by one-way analysis of variance (ANOVA), followed by Tukey or Games-Howell test. Values of *p* < 0.05 were considered statistically significant. Statistics were processed using the SPSS statistics 16.0 (SPSS Inc., USA).

## Results

### HPLC Fingerprint of Gps

**Fig. 1:**
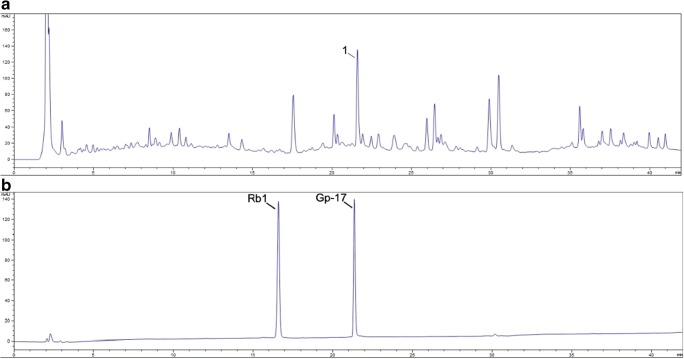
HPLC fingerprint of Gps. High performance liquid chromatography fingerprint profile of gypenosides and standard substances (gypenoside-17 and ginsenoside Rb1). **a** Peak 1 presented a substance that has a similar structural characteristic as Gp-17

The HPLC fingerprint of Gp is shown in Fig. 1a, and was composed of a series of distinct characteristic peaks. The HPLC analysis result of the two standard substances, Gp-17 and Ginsenoside Rb1, which are structurally different, is shown in Fig. 1b. Peak 1 in Fig. 1a appears to correspond to Gp-17.

### Gp Protects RGCs from H_2_O_2_-Induced Insults

**Fig. 2:**
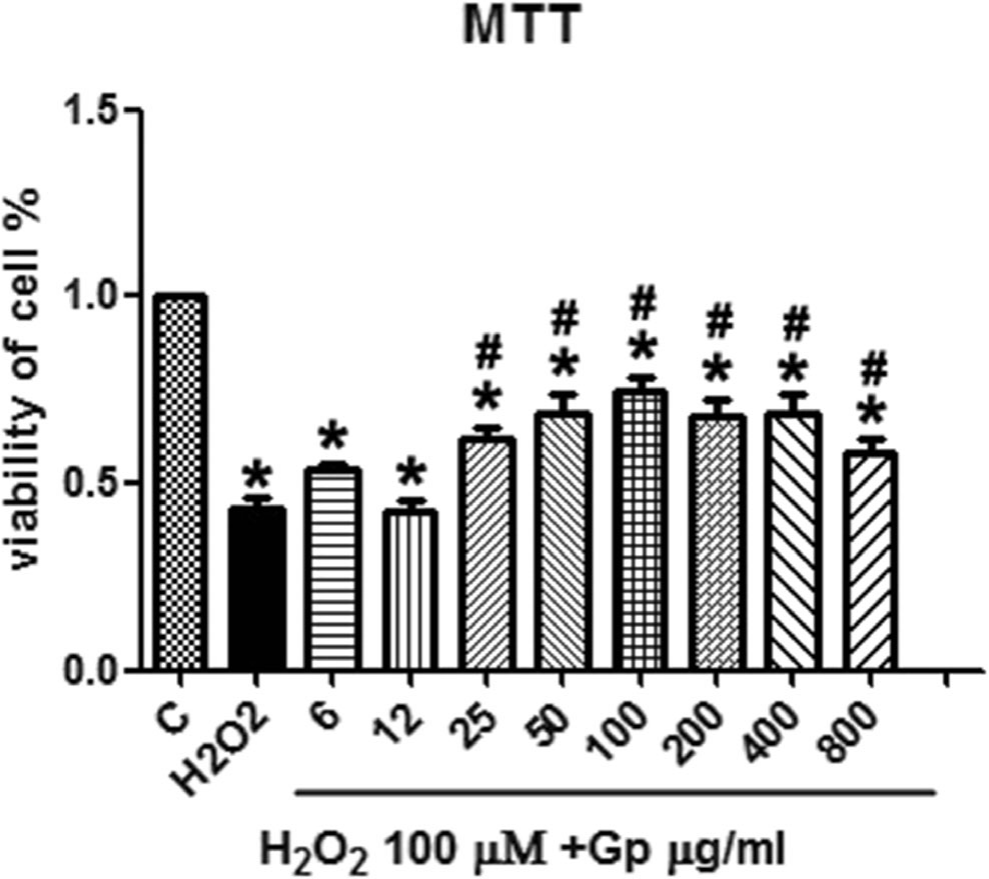
Retinal ganglion cell (RGC) viability in the presence of H_2_O_2_. Viability of RGCs was quantitated in the presence of H_2_O_2,_ and the protective effects of Gp on RGCs undergoing oxidative injury was quantitated by the MTT assay. ^*^p < 0.05 when compared with the control group; ^#^*p* < 0.05 compared with the H_2_O_2_ group. Data were presented as mean ± SE (*n* = 3)

To evaluate whether Gp could protect RGCs against H_2_O_2_ damage, we measured cell viability by the MTT assay. After H_2_O_2_ incubation, the model group’s RGC survival rate (without Gp) was reduced to 43.38 ± 8.72%. Survival rates of the Gp-treated group were 69.09 ± 14.94%, 74.39 ± 12.36%, and 68.26 ± 12.75% at final concentrations of 50, 100, or 200 μg/ml. Relative to the control group, treatment with H_2_O_2_ significantly decreased the viability of RGCs (*p* < 0.05, Fig. 2), and Gp treatment at 50, 100, or 200 μg/mL significantly increased RGC viability relative to that in the H_2_O_2_ treatment group (*p* < 0.05; Fig. 2).

### Gp Reverses H_2_O_2_-Induced Intracellular ROS

**Fig. 3:**
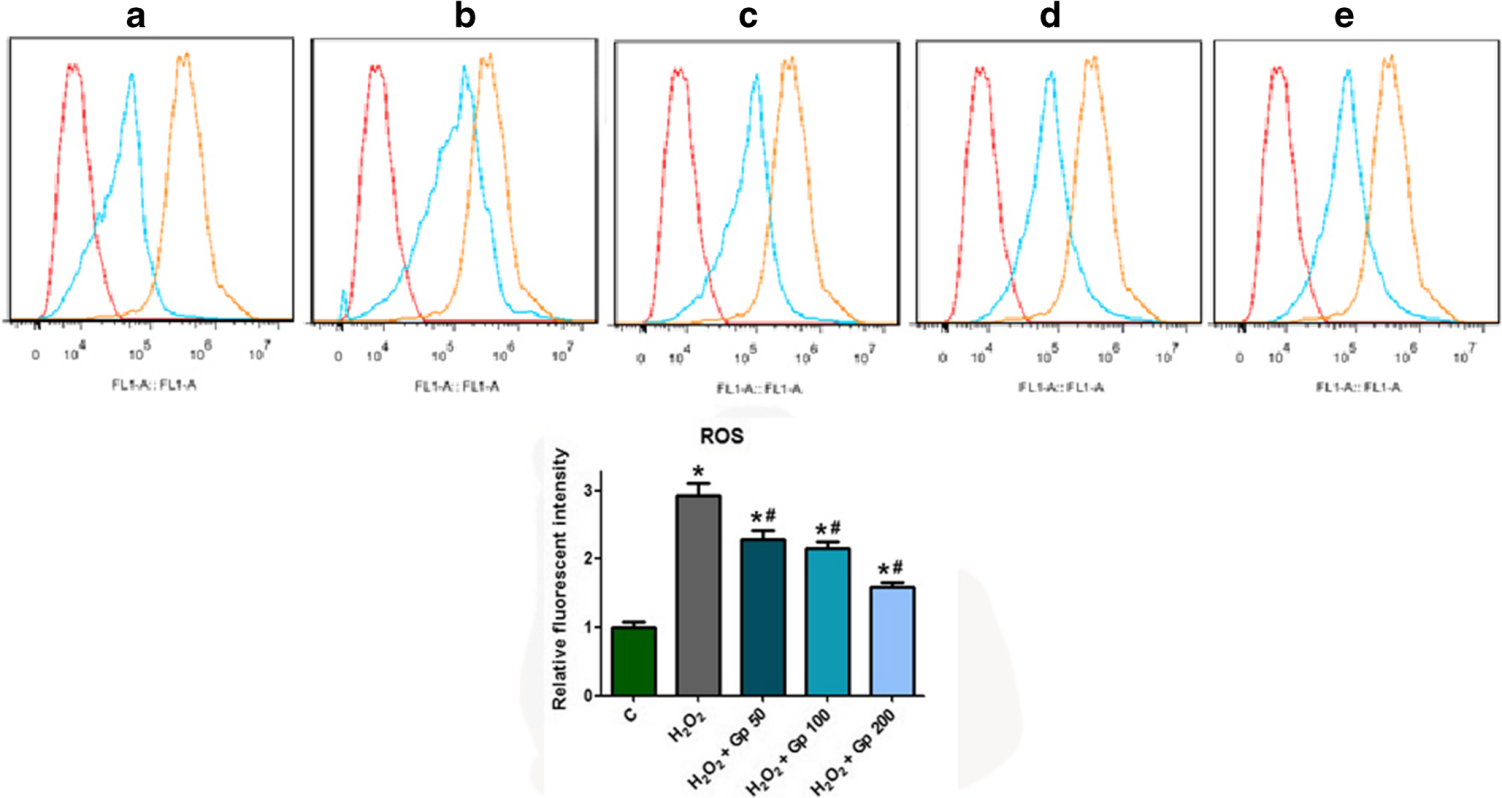
Quantitation of ROS in control and experimental groups. ROS was quantitated in retinal ganglion cells (RGCs) using cell cytometry. **a** Normal control. **b** H_2_O_2_ treated. **c**–**e** Increasing concentrations of Gp. In the cytometry data shown in panels **a**–**e**, negative control samples are the red curve, positive controls are the yellow curve, and the experimental sample is shown by the blue curve. ^*^p < 0.05 when compared with the control group; ^#^*p* < 0.05 when compared with the H_2_O_2_ group. Data were presented as mean ± SE (*n* = 3)

The change in intracellular ROS levels in the H_2_O_2_ and/or Gp-treated cells was measured by flow cytometry. Compared with control group, the level of intracellular ROS was increased in RGCs after H_2_O_2_ treatment (*p* < 0.05; Fig. 3). ROS levels in H_2_O_2_-treated RGCs increased significantly, by approximately 3-fold. Gp treatment in all of the concentrations decreased H_2_O_2_-induced ROS levels by approximately 22.18–45.73%, respectively. The high concentration of 200 μg/ml works best, manifesting the similar protective effects in a dose-dependent manner.

### Gp Protects RGCs Against Apoptosis Induced by H_2_O_2_

**Fig. 4:**
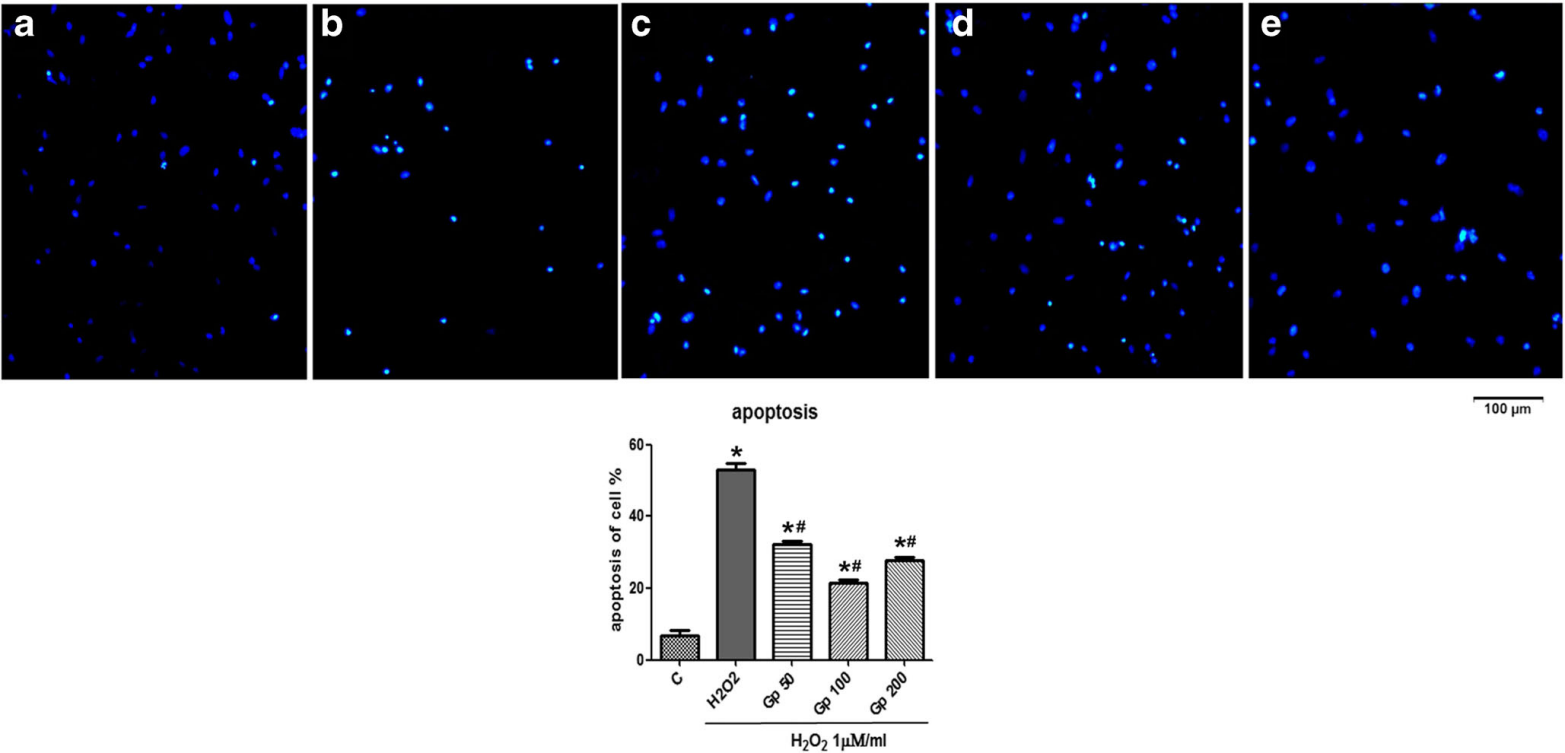
Retinal ganglion cell (RGC) cultures were stained with Hoechst 33342 stain to visualize nuclei. In control cultures, the nucleus was slightly and uniformly stained. Cells undergoing apoptosis showed high-density fluorescence, karyopyknosis, and nuclear fragmentation. **a** Normal control. **b** H_2_O_2_ treated. **c**–**e** Increasing concentrations of Gp. ^*^p < 0.05 when compared with the control group; ^#^*p* < 0.05 when compared with the H_2_O_2_ group. Data were presented as mean ± SE (*n* = 3)

**Fig. 5:**
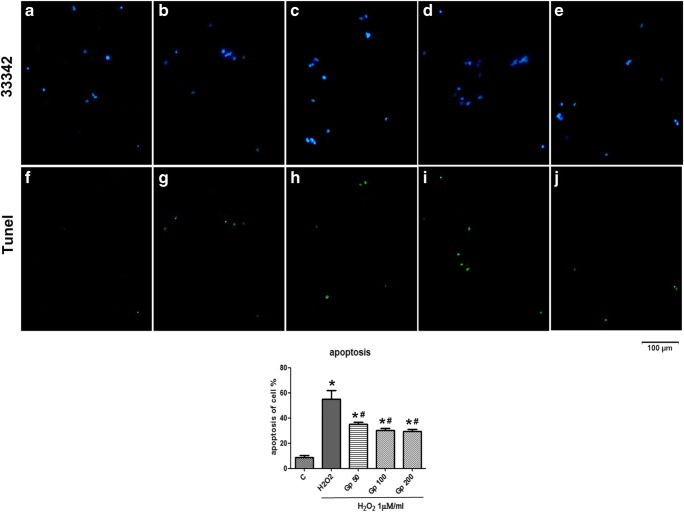
Nuclei were stained with Hoechst 33342 (panels **a**–**e**) and apoptosis was detected by TUNEL analyses of the same field is shown in panels **f**–**j**. A/F normal control, B/G H_2_O_2_ treated, C/H treatment with 50 Gp, D/I treatment with 100 Gp, and E/J treatment with 200 Gp. ^*^p < 0.05 when compared with the control group; ^#^*p* < 0.05 when compared with the H_2_O_2_ group. Data were presented as mean ± SE (*n* = 3)

Apoptosis in RGCs was detected using Hoechst 33342 and TUNEL staining. As shown in Figs. 4 and 5, transient treatment with 100 μM H_2_O_2_ for 4 h increased the number of cells stained for cellular pyknotic nuclei (53.11 ± 2.74%) compared with the control group (6.65 ± 2.68%). However, treatment with 50–200/mg of Gp reduced the percentage of Hoechst 33342-positive apoptotic cells to 32.37 ± 1.26% (*p* < 0.05) and 21.53 ± 1.32% (*p* < 0.05), respectively (Fig. 4). The protective effects of Gp on apoptosis in RGCs were further confirmed by TUNEL staining, and a similar result was observed. The ratio of TUNEL positive apoptotic cells in H_2_O_2_ group increased to 55.18 ± 11.90% when compared with a control (8.72 ± 2.83%, *p* < 0.05). After treatment with 50–200 Gp, RGCs became more resistant to H_2_O_2_-induced oxidative stress injury; the ratio decreased to 35.24 ± 2.38%, 30.35 ± 2.94%, and 29.60 ± 2.50% (Fig. 5). Gp treatment markedly reduced the loss of RGCs due to H_2_O_2_-induced apoptosis.

### Effect of Gp on mRNA Expression of iNOS, COX-2, Nrf-2, HO-1, and Gpx1/2 in RGCs

**Fig. 6:**
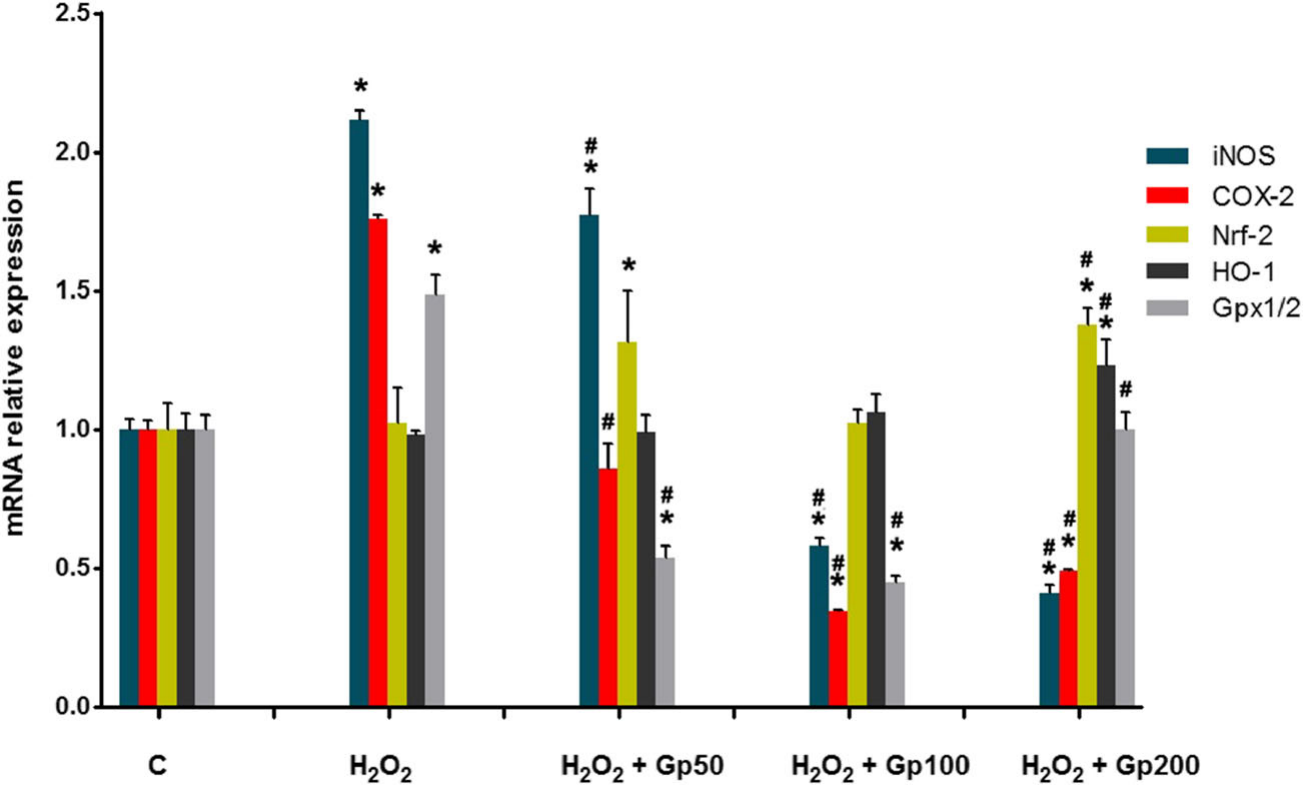
The relative mRNA expression of iNOS, COX-2, Nrf-2, HO-1, and Gpx1/2 was detected by quantitative real-time PCR. C denotes control retinal ganglion cell (RGC) cultures. ^*^p < 0.05 when compared with the control group; ^#^*p* < 0.05 when compared with the H_2_O_2_ group. Data were presented as mean ± SE (*n* = 3)

We investigated the effects of Gp on H_2_O_2_-induced expression of the inflammatory mediators iNOS, COX-2, Nrf-2, HO-1, Gpx1/2, and apoptosis-associated proteins Bcl-2, Bax, and caspase-3 genes using real-time RT-PCR. As shown in Fig. 6, Gp raised iNOS, COX-2, and GPx1/2 expressions compared with the control cells; the mRNA expression of COX-2 and iNOS was significantly inhibited by Gp in all concentrations assayed. Expression of Nrf-2 and OH-1 genes was promoted by Gp in medium and high concentrations (Fig. 6).

### Effect of Gp on mRNA Expression of Bcl-2, Bax, and Caspase-3

**Fig. 7:**
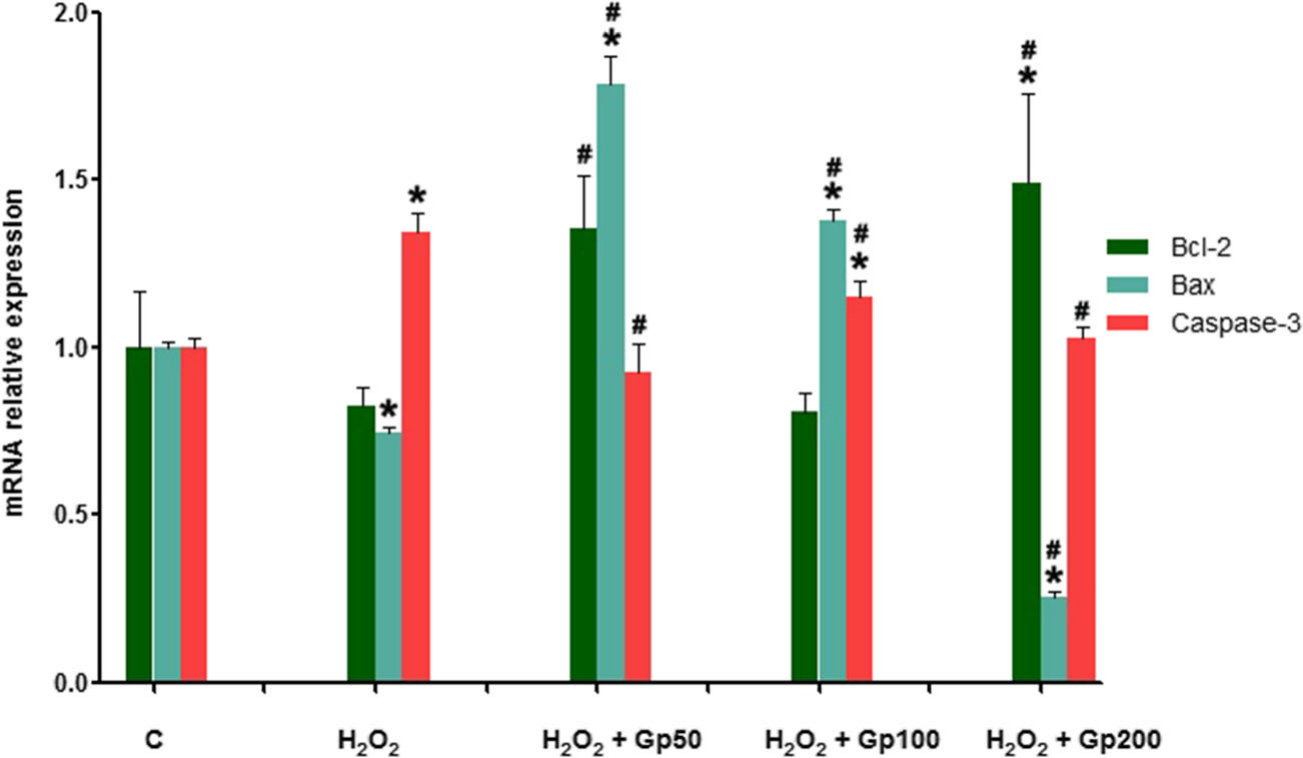
The relative mRNA expressions of Bcl-2, Bax, and caspase-3 were detected by quantitative real-time PCR. ^*^p < 0.05 when compared with the control group; ^#^*p* < 0.05 when compared with the H_2_O_2_ group. Data were presented as mean ± SE (*n* = 3)

Cells treated with H_2_O_2_ had a lower level of Bax mRNA expression and higher caspase-3 expression. Compared with this Bcl-2 expressions was increased in the presence of low and high concentrations of Gp. Bax expression was increased in the low and medium concentrations of Gp but decreased when treated with high concentrations of Gp. All concentrations of Gp inhibited the expression of caspase-3 (Fig. 7).

### Effect of Gp on Protein Expression of iNOS, COX-2, Nrf-2, HO-1, and Gpx1/2 in RGCs

**Fig. 8:**
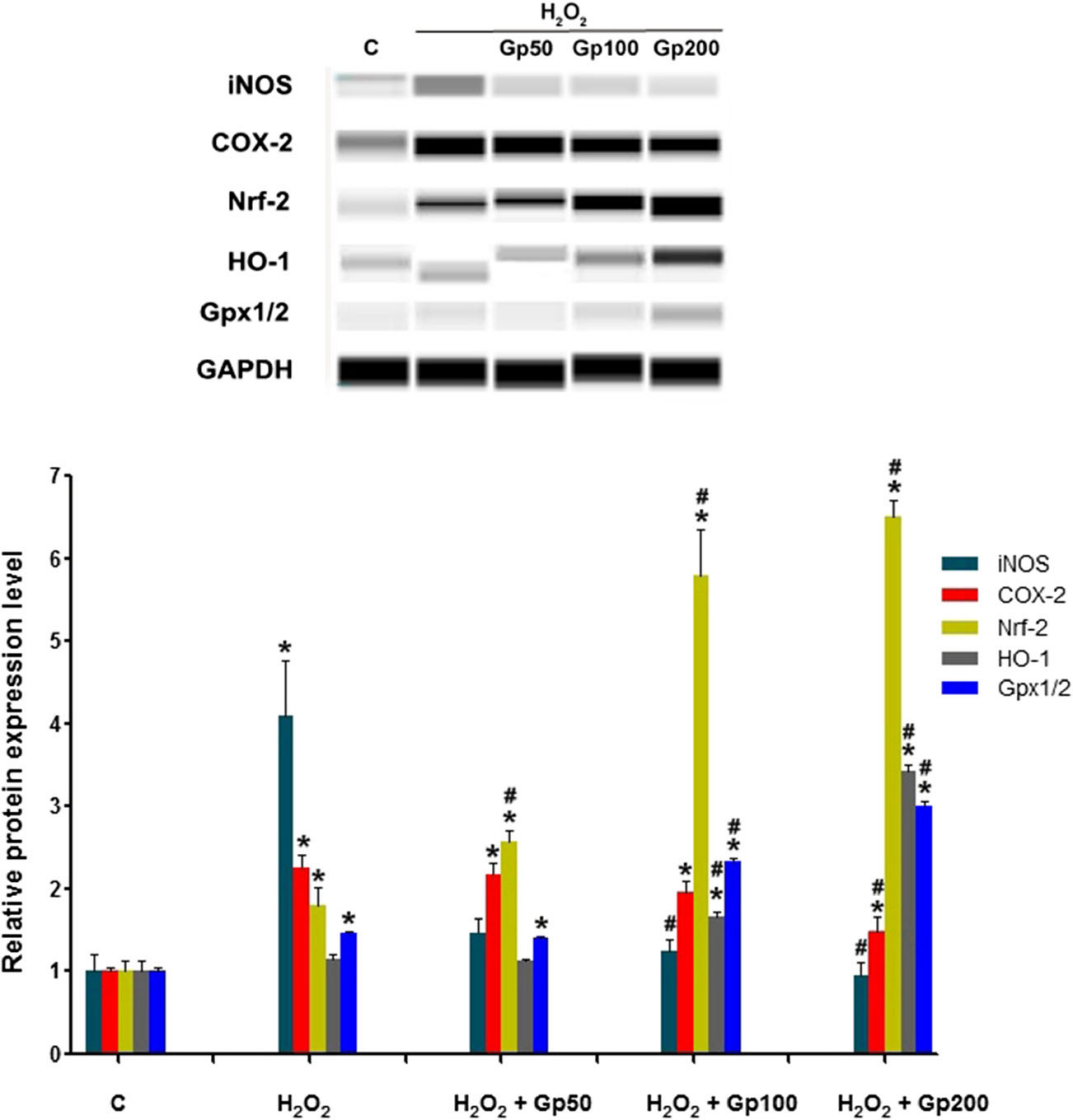
The relative protein expression of iNOS, COX-2, Nrf-2, HO-1, and Gpx1/2 was detected by protein immunoblotting. ^*^p < 0.05 when compared with the control group; ^#^*p* < 0.05 when compared with the H_2_O_2_ group. Data were presented as mean ± SE (*n* = 3)

Protein immunoblotting analysis demonstrated that COX-2 and iNOS were significantly upregulated in the H_2_O_2_-treated group when compared with control. Gp resulted in a significant decrease in both the expression of Cox-2 and iNOS in the high concentration of Gp (about 1/3-fold) administered after incubation with H_2_O_2_ (Fig. 8). Further, the levels of expression of Nrf-2, a transcription factor known to play a role in the expression of survival genes related to oxidative stress, were significantly increased from 43 to 264.46% in all concentration Gp treatment, and robustly medium and high concentrations (about 2.2–2.6-fold). In medium and high concentration Gp, the levels of expression of OH-1 and Gpx1/2 were significantly increased when compared with H_2_O_2_ treatment (Fig. 8).

### Effect of Gp on Protein Expression of Bcl-2, Bax, and Caspase-3

**Fig. 9:**
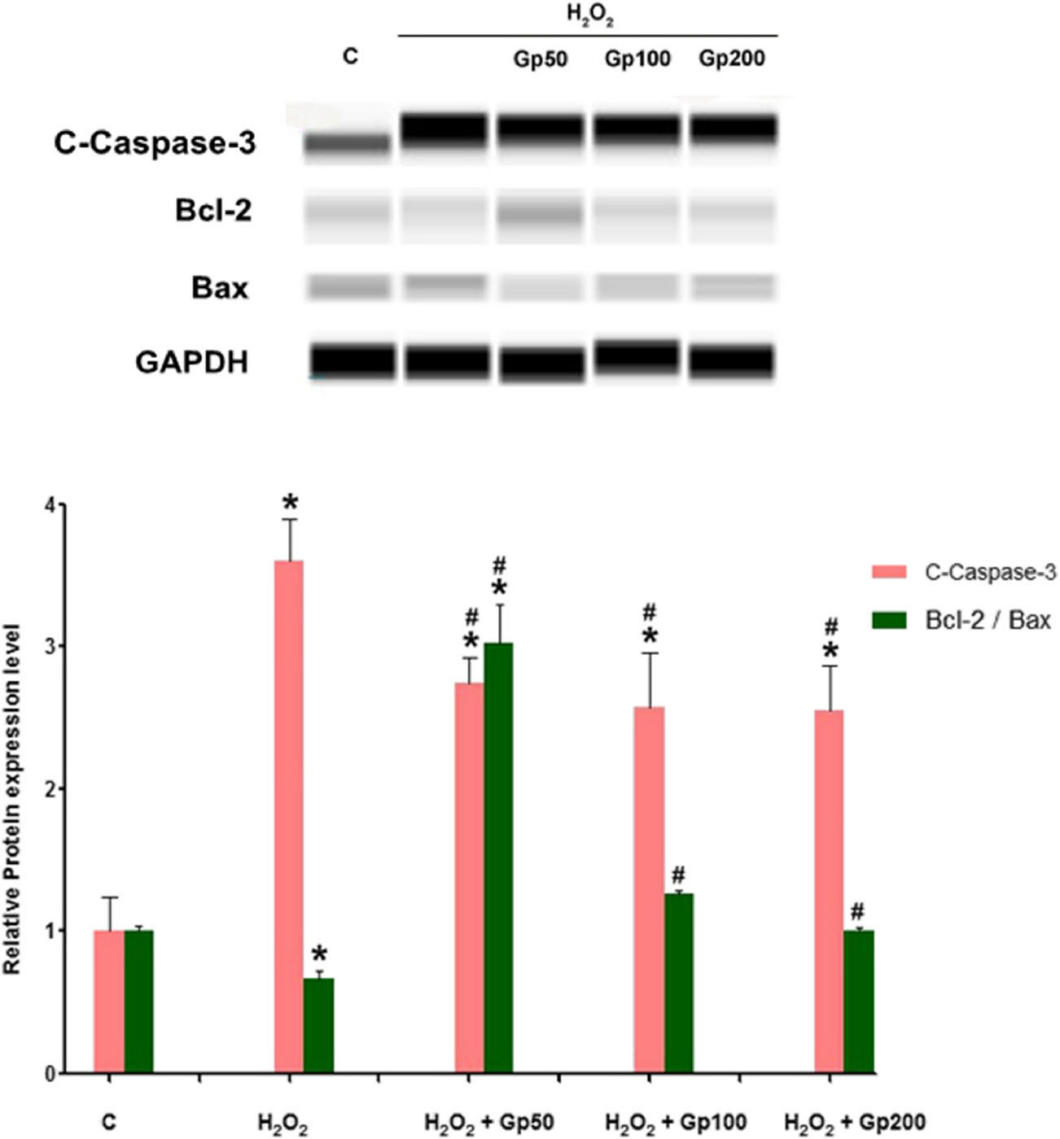
The relative protein expressions of Bcl-2, Bax, and cleaved caspase-3 were detected by protein immunoblotting. ^*^p < 0.05 when compared with the control group; ^#^*p* < 0.05 when compared with the H_2_O_2_ group. Data were presented as mean ± SE (*n* = 3)

The protein expression of Bax, Bcl-2, and caspase-3 in the RGCs is shown in Fig. 9. The expression of caspase-3 protein in the H_2_O_2_ treatment group was significantly higher, and the ratio of Bcl-2/Bax protein expression was lower than that in control group and treatment groups (*p* < 0.05). However, the ratio of Bcl-2/Bax protein expression in the RGCs was significantly increased in the Gp treatment groups compared with the H_2_O_2_ treatment group (*p* < 0.05) (Fig. 9).

### Gp Prevents the H_2_O_2_-Mediated Inhibition of the PI3-K/Akt Activation

**Fig. 10:**
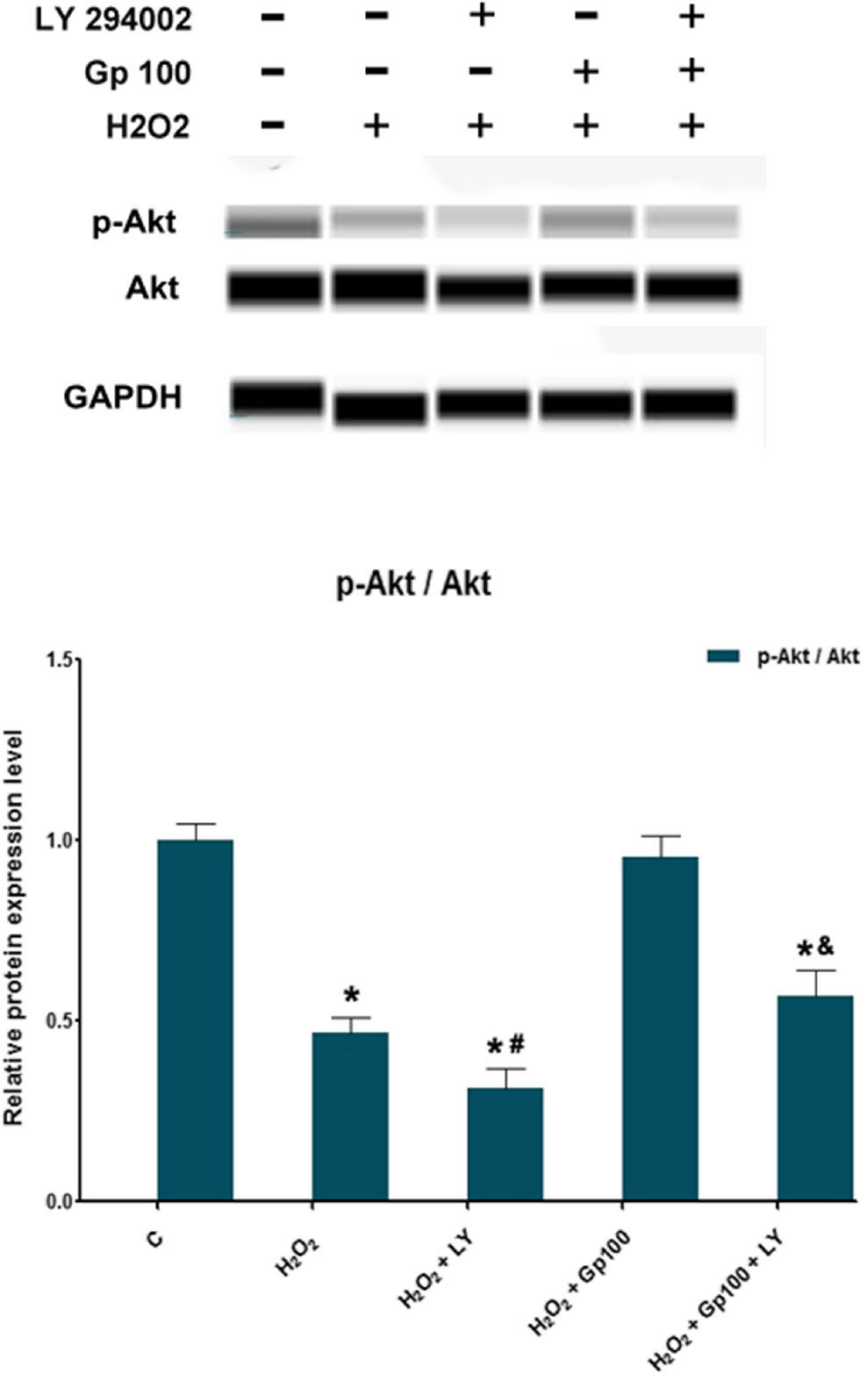
The relative protein expression of p-Akt/Akt was detected by protein immunoblotting. ^*^p < 0.05 when compared with the control group; ^#^*p* < 0.05 when compared with the H_2_O_2_ group. ^&^*p* < 0.05 when compared with the gypenoside treatment group. Data were presented as mean ± SE (*n* = 3)

As shown in Fig. 10, H_2_O_2_ decreased the phosphorylation of Akt, but Gp significantly stimulated the phosphorylation of Akt. This activation was inhibited significantly by the pre-incubation with a PI3K inhibitor LY294002.

## Discussion

Recent data suggest that oxidative stress plays an important role in inflammatory demyelinating disease. Inflammation causes the significant production of ROS, which results in the injury of cellular DNA, mitochondrial proteins, and membrane lipids. The pathophysiology of MS is complex and not completely understood. It involves redox, inflammatory/autoimmune, and neurodegenerative components, all of which are important (Miljkovic and Spasojevic 2013). At the chronic phase of MS, even in the absence of peripheral immune cells, oxidative stress caused by astrocytes and microglia alone explains the ongoing subclinical neuronal dysfunction (Radbruch et al. 2016). Therefore, antioxidant treatment is considered to be a viable strategy for the treatment of inflammatory demyelinating diseases. Administration of antioxidants can reduce injury in an animal model of demyelination and MS (Binyamin et al. 2015; Miller et al. 2019).

In a previous study, we evaluated the protective effect of Gp in a mouse model of experimental autoimmune optic neuritis. Treatment with Gp exerted protective effects on retinal nerve fibers and axons. These results strongly indicate that the Gp might be a useful supplement for optic neuritis treatment (Zhang et al. 2017).

There have been extensive studies reporting on the neuroprotective effects of triterpenoid saponins (Biswas and Dwivedi 2019; Sun et al. 2015). The total saponins from Gynostemma pentaphyllum used in this study were a mixture with a purity of ≥ 98%. The pathological disease processes are complicated, and there may be multiple pathways underlying the pharmacodynamics of the drug. A mixture of multiple compounds may exert synergistic effects; such observations demonstrate the advantages of multitarget therapy. However, the neuroprotective mechanisms, pathways, upstream, and downstream molecules of Gp, as well as the presence of drug-drug interactions, remain unclear. It is currently believed that the possible mechanisms of Gp include antioxidation, anti-apoptosis, anti-inflammation, regulation of neural networks, and nutritional factors (Sun et al. 2015; Li et al. 2014; Yang et al. 2013; Shin et al. 2014; Wang et al. 2010; Zhao et al. 2017). Our previous study found that Gp exhibits neuroprotective effects in an animal model of experimental autoimmune optic neuritis (EAON). EAON-induced injuries include tissue damage due to inflammation (oxidative damage), activation of neuronal apoptosis, and changes in the extracellular environment (activation and proliferation of glial cells, increased levels of inflammatory factors, and reduced levels of growth factors). The antioxidative, neuroprotective, and anti-inflammatory effects of Gp may contribute to multitarget interventions against EAON, but the specific mechanisms require further investigation.

In the present study, we demonstrated the antioxidant, anti-apoptosis, and anti-inflammatory effects of Gp in a H_2_O_2_-insult model in RGCs for the first time. We examined cell viability under conditions of oxidative stress and found that 50–200 μM Gp significantly increased the viability of RGC cultures exposed to H_2_O_2_. Our results were consistent with previous studies, which show that Gp reduced ROS levels during ischemia-reperfusion injury, and protect RGCs from oxidative stress in a concentration-dependent manner (Yu et al. 2016a).

Based on the results of MTT, cells treated with 25-500 μg/ml of Gp showed higher viability than the H_2_O_2_ group cells, but there was no improving tendency in the cell viability alongside increasing concentrations of Gp, whose effects seem to saturate. Based on the ROS level in H_2_O_2_-treated cells, 50-200 μg/ml of Gp reduced the level of ROS after oxidative stress, indicating that it exhibits antioxidative effects. The numerical results of ROS level seem to show a declining tendency with increasing concentrations of Gp, but the enhanced protective effects of Gp at higher concentrations were not observed in the MTT results. We know that H_2_O_2_ also induces antioxidative responses while causing damage to cells (Kregel 2002). Therefore, the preconditioning of cells with H_2_O_2_ increases the tolerance of cells to oxidative stress. However, our experimental results were observed 4 h after being exposed to oxidative stress. Given the results of previous studies on oxidative preconditioning of cells (Plaks et al. 2004), it is difficult to explain the adaptive responses of RGC to the stress within such a short period of time. Therefore, we speculate that RGCs were not preconditioned to H_2_O_2_-induced stress in our study. The protective effects of Gp on RGC did not increase significantly with the increase in its concentration, indicating that the saturation trend may be related to the saturation of the drug in cells or the saturation of cell-surface receptors that recognize the drug. Hence, more extensive studies are required to determine whether a higher concentration of Gp yields greater cell viability and reduces the production of ROS, and whether a 4-h exposure to oxidative stress leads to the oxidative preconditioning of RGC. Besides, the mechanisms by which Gp enters cells and affects RGC also require further investigation.

The inflammatory environment may lead to the generation of oxygen- and nitrogen-free radicals as well as proinflammatory cytokines that in turn exacerbate the inflammatory response. Proinflammatory cytokines are released by activated glial cells and include inducible nitric oxide synthase (iNOS) and cyclooxygenase-2 (COX-2) which are important pathologic factors in MS (Ortiz et al. 2013). In general, inflammatory stimuli are known to activate the expression of iNOS and COX-2 in activated macrophages. iNOS is involved in the production of NO and has been found to be upregulated in active lesion areas in multiple sclerosis (Cross et al. 1998). Some investigations showed that Gp exhibits an anti-inflammatory effect by inhibiting iNOS enzymatic activity or decreasing the expression levels of iNOS (Lin et al. 1993; Aktan et al. 2003; Cai et al. 2016). Here, we investigated the effect of Gp on expressions of iNOS and COX-2 in oxidative stress–mediated cell damage. Our results indicate that treatment with H_2_O_2_ induced the overexpression of iNOS and COX-2 at the mRNA and protein levels. Gp treatment, especially in high concentration, appreciably alleviates the H_2_O_2_-mediated iNOS and COX-2 overexpression both at the mRNA and protein level and is in line with its anti-inflammatory effect. This result was consistent with our previous study on EAON, an animal model of MS, demonstrating that there was less inflammatory reaction in the Gp treatment groups (reference). Therefore, the anti-inflammatory effect of Gp may be attributable to its inhibition of iNOS and COX-2.

Furthermore, gypenosides also influence nuclear transcription factors such as Nrf-2. Nrf-2 is a critical transcription factor that regulates antioxidant genes by binding to antioxidant response elements (AREs). Nrf-2 regulates the gene expression of many protective antioxidant and detoxification enzymes such as HO-1 and GPx. One study shows that the activation of Nrf-2 may attenuate the pathogenesis of experimental autoimmune encephalomyelitis (EAE), an animal model of MS (Johnson et al. 2010). The neuroprotective effect of OH-1 in EAE has been proved, as the induction of HO-1 after EAE onset showed a therapeutic effect in both relapsing-remitting and chronic EAE (Chakrabarty et al. 2003). Thus, HO-1 induction may be beneficial in the initial stages of the disease (Janssen et al. 2015).

The families of natural triterpenoids have recently emerged as potent regulators of the Nrf2/ARE pathway. A natural pentacyclic triterpene, oleanolic acid, exerts neuroprotective effects directly through Nrf2-dependent induction of antioxidant gene in a murine model of MS (Pareek et al. 2011). Recent research reported another example of a triterpenoid named tenuigenin, which inhibits the LPS-induced inflammatory cytokine production and upregulated the expression of Nrf2 and HO-1 in an LPS-induced mouse model of memory deficit (Lu et al. 2017).

Gp, the predominant components of *Gynostemma pentaphyllum*, are dammarane tetracyclic triterpenoids. In our study, we found that in the model of H_2_O_2_-induced oxidative stress, Gp have the antioxidant capacity to regulate Nrf-2 and OH-1 activation. Meanwhile, the antioxidation effect of Gp in the present study also increases the content of glutathione peroxidase-1 (GPx) to strengthen the endogenous antioxidant defense system. GPx-1 is an antioxidant enzyme that limits hydrogen peroxide accumulation to alleviate its harmful effects in the cell.

Hence, the possible mechanisms of the antioxidant action of Gp include direct binding with the free radicals to stop the chain reaction of ROS generation, inhibit proinflammatory cytokines such as iNOS and COX-2, and promote the activity of the antioxidant system by increasing Nrf-2 transcriptional activation to promote antioxidant enzymes.

MS is a chronic inflammatory demyelinating disease of the central nervous system and is associated with the formation of focal myelin loss and progressive neurodegeneration (Mahad et al. 2015). Reactive oxygen and nitrogen species, produced by activated microglia and macrophages, cross membranes and compete with oxygen to decrease respiratory chain function (Haider et al. 2015). In addition, the specific molecular characteristics of mitochondria (both mtDNA and proteins are particularly susceptible to oxidative damage) make it a compartment which is highly vulnerable to the impact of ROS (Apostolova and Victor 2015). In patients with multiple sclerosis, mitochondrial dysfunction has been described extensively in the cortex and white matter (Sadeghian et al. 2016). Mitochondria regulate energy generation, cellular calcium homeostasis, and represent a physical point of convergence for many apoptosis inducing signals in mammalian cells (Bhola and Letai 2016).

The Bcl-2 family of proteins controls a critical step in commitment to apoptosis by regulating permeabilization of the mitochondrial outer membrane (Shamas-Din et al. 2013). The Bcl-2 family is an anti-apoptotic protein and inhibits cell endoplasmic reticulum Ca2+ release, lipid peroxide formation, and free radical production. By contrast, another member of Bcl-2 family, Bax, is referred to as a pro-apoptotic effector protein and is required for mitochondrial-mediated apoptosis (Renault et al. 2013). The caspases are a family of cysteine proteases, which act as executioners during apoptosis, with caspase-3 being an important protein in this family (Slee et al. 2001).

In the present study, Gp displayed neuroprotective effects by increased RGC viability and alleviating H_2_O_2_-induced oxidative stress injury resulting in RGC apoptosis. Our findings showed that Gp inhibited H_2_O_2_-mediated RGC apoptosis meanwhile increasing the ratio of Bcl-2 to Bax and controlling cleaved caspase-3 activation. These results suggest that the neuroprotective effects of Gp may be mediated by the alleviation of mitochondrial-mediated apoptosis.

We studied the antioxidative effects of Gp on RGC, and found that the Gp mixture had protective effects on RGC against oxidative damage. Specifically, the antioxidative effect of Gp can reduce the production of ROS, increase the expressions of Nrf-2/ARE and OH-1, reduce the production of inflammatory factors iNOS and COX-2, and reduce apoptosis via the endogenous apoptotic pathway. Taken together with previous studies on Gp (Shang et al. 2006; Alhasani et al. 2018; Yu et al. 2016b; Yang et al. 2017), we believe that this antioxidative effect may be directly associated with ROS clearance, improvement of endogenous antioxidant capacity, and reduced levels of inflammatory factors and mitochondrial damage, thereby reducing the rate of apoptosis. However, its specific mechanisms still require extensive study. As for single ingredients in Gp, GP-17 exerts anti-apoptotic and antioxidative effects via the estrogen receptor-mediated PI3k pathway (Yang et al. 2017; Meng et al. 2014). We observed that Gp activates PI3K and can be inhibited by blockers (LY294002). Thus, based on previous studies, we speculate that the PI3k/AKT pathway may play a role in protective effect of Gp against H_2_O_2_-induced cell damage by inducing Nrf2/HO-1 expression. However, whether the protective effect of Gp in H_2_O_2_-insulted RGCs is via the PI3k/Akt pathway that needs to be further elucidated, and the antioxidant and anti-apoptosis effects of Gp require further in vivo investigation.

In summary, this study has shown that Gp attenuates H_2_O_2_-induced oxidative damage in RGCs by increasing cell viability and reducing apoptosis. This neuroprotective effect is coincident with the depression of the mitochondria apoptosis pathway, and alleviation of intracellular oxidative damage and inflammatory response. Massive studies—such as high-throughput screening, transcriptomic, and metabolomic studies—are still required to determine the component(s) of the saponins that are effective, the presence of drug-drug interactions, the mode, the site of action, and underlying pathways.
